# Progressive Muscle Relaxation Improves Anxiety and Depression of Pulmonary Arterial Hypertension Patients

**DOI:** 10.1155/2015/792895

**Published:** 2015-04-01

**Authors:** Yunping Li, Ranran Wang, Jingqun Tang, Chen Chen, Ling Tan, Zhongshi Wu, Fenglei Yu, Xiang Wang

**Affiliations:** ^1^State Key Laboratory of Medical Genetics, The Second Xiangya Hospital, Central South University, Changsha 410011, China; ^2^Department of Thoracic Surgery, The Second Xiangya Hospital, Central South University, Changsha 410011, China; ^3^Department of Cardiovascular Surgery, The Second Xiangya Hospital, Central South University, Changsha 410011, China; ^4^Cancer Research Institute, Central South University, Changsha 410011, China; ^5^Key Laboratory of Carcinogenesis and Cancer Invasion, Ministry of Education, Changsha 410011, China; ^6^Key Laboratory of Carcinogenesis, Ministry of Health, Changsha 410011, China

## Abstract

We explored the effects of progressive muscle relaxation (PMR) on anxiety, depression, and quality of life (QOL) in patients with pulmonary arterial hypertension (PAH). One hundred and thirty Han Chinese patients with PAH were randomly assigned to a PMR group (*n* = 65) and a control group (*n* = 65). In a 12-week study duration, the PMR group received hospital-based group and in-home PMR practice, while the control group received hospital-based mild group stretching and balance exercises. The control group and the PMR group were comparable at baseline. After 12 weeks of intervention, the PMR group showed significant improvement in anxiety, depression, overall QOL, and the mental component summary score of QOL (*P* < 0.05) but not the physical component summary score of QOL or the 6-minute walking distance. In contrast, the control group showed no significant improvement in any of the variables. Moreover, the PMR group showed significant improvement in all QOL mental health domains (*P* < 0.05) but not the physical health domains. In contrast, the control group showed no significant improvement in any QOL domain. In conclusion, this study suggests that PMR practice is effective in improving anxiety, depression, and the mental health components of QOL in patients with PAH.

## 1. Introduction

Pulmonary arterial hypertension (PAH) is a progressive and debilitating chronic illness caused by increased pulmonary vascular resistance with progressive right ventricular dysfunction [1]. Many mechanisms can lead to the development of PAH, including idiopathic diseases, connective tissue diseases, and congenital heart diseases [1]. At the time of diagnosis, patients with PAH are usually severely affected with impaired exercise capacity and shortness of breath due to elevated pulmonary artery pressure, increased pulmonary vascular resistance, and right heart failure [2–4]. In consequence, the patients have to manage various life stressors, such as physical burdens, unclear prognosis, high cost of treatment, and often unemployment, which can have a psychological impact and may lead to the development of mental health problems such as depression and anxiety [5, 6]. Depression has been detected in up to 55% of patients with PAH [7]. A recent study has shown that anxiety and depression are frequently detected and significantly correlated with quality of life (QOL) in patients with PAH [8].

Approximately 24.1% of patients with pulmonary hypertension and mental health problems received psychopharmacological treatment [5]. Although pharmacotherapy can successfully treat anxiety and depression, psychiatric medications alone or through their interaction with other drugs can produce side effects, and some patients are unwilling to take psychiatric medications. Some patients may be reluctant to take any additional drugs, perceiving that as a sign of loss of control and personal weakness in the handling of their illness [9]. Relaxation techniques have been shown as an effective adjunctive therapy for anxiety and depression, providing patients with self-maintenance coping skills to reduce anxiety symptoms [10–15]. Progressive muscle relaxation (PMR) is a systematic technique used to achieve a deep state of relaxation and has been shown to improve health-related QOL in a variety of medical and psychiatric illnesses [16–18]. In the present study, we for the first time explored the effects of PMR as a psychosomatic intervention in anxiety, depression, and QOL in patients with PAH.

## 2. Materials and Methods

### 2.1. Patients

**Figure 1:**
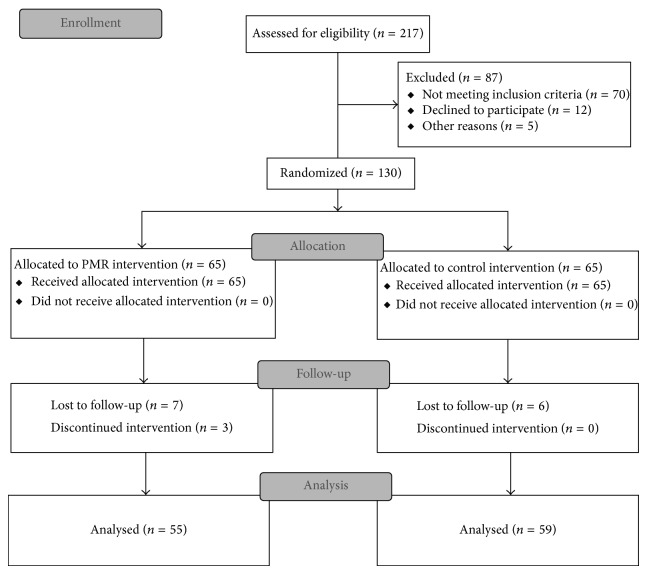
Flow chart of patient enrollment.

From August 2012 to September 2013, 130 Han Chinese patients with PAH (40 males and 90 females), aged 25–80 years, were recruited in this randomized controlled study at the Second Xiangya Hospital, Central South University. The inclusion criteria were as follows: (1) being diagnosed with PAH by international guidelines [19]; (2) having been stable under optimized PH-targeted medical treatment for at least 2 months; (3) being of WHO functional classes I–III [2]; (4) having an above-elementary school education; and (5) being able to give informed consent. The exclusion criteria were as follows: (1) having a family or personal history of mental illness; (2) having concurrent oncologic or psychiatric diseases; (3) being currently under treatments for anxiety or depression; and (4) being of WHO functional class IV [2]. A total of 217 consecutive patients who met the criteria were interviewed, out of which 130 agreed to participate in the study. The patients were assigned to a PMR group (*n* = 65) and a control group (*n* = 65) using block randomization with randomly selected block sizes of 4, 8, and 10 and an allocation ratio of 1 : 1. Based on a two-tailed *α* = 0.05, power = 0.80, and an effect size = 0.50 (based on data from a pilot study with 50 patients with PAH), a sample size of 51 was originally calculated for comparison of means between the two groups [20]. Taking into account an anticipated 20% dropout rate, 65 patients were selected for each group to ensure an adequate final sample size. At the end of study, 114 effective cases were collected, with 55 cases in the PMR group and 59 cases in the control group. The reasons for the patients' dropout included the following: (1) moving out of town (*n* = 1, control group; *n* = 0, PMR group); (2) feeling unnecessary to continue PMR practice because of lack of improvement (*n* = 3, PMR group); and (3) loss of contact (*n* = 5, control group; *n* = 7, PMR group). The patient enrollment flow chart is shown in Figure 1. The study was approved by the Ethics Committee of the Second Xiangya Hospital, Central South University. Written informed consent was obtained from all participating patients before the start of the study.

### 2.2. Instruments

All questionnaires originally developed in English were translated into Chinese and then back-translated to ensure accurate translation of the measures. Anxiety and depression were assessed using the Hospital Anxiety and Depression Scale (HADS), a validated and widely used tool for assessing psychological distress in medical patients [21]. It comprises 14 items that tap anxiety (seven items; score range 0–21) and depression (seven items; score range 0–21), and higher scores correspond to greater distress [21]. The HADS has been found to have good internal consistency, reliability, and validity in a variety of medical populations [21, 22]. It also allows longitudinal assessment with repeated testing at intervals of 1 week or more and is sensitive to changes in a patient's emotional state [22]. The Cronbach's *α* was 0.83 for HADS-Anxiety and 0.86 for HADS-Depression in the present study. Health-related QOL was measured with the SF-36 scale, which comprises 36 items covering eight domains: physical function (10 items), role limitation caused by physical problems (4 items), body pain (2 items), general health perception (5 items), vitality-energy (4 items), social function (2 items), role limitations caused by emotional problems (3 items), and mental health (5 items) [23]. The scores of the QOL physical health domains (physical function, physical role, bodily pain, and general health) can be summarized into a physical component summary score (QOL-PCS), and the scores of the mental health domains (vitality, social function, emotional role, and mental health) can be summarized into a mental component summary score (QOL-MCS). Higher score indicates better QOL. Test-retest correlation coefficient of the scale reportedly is 0.80 with a 2-week interval [23]. Cronbach's alpha of SF-36 was 0.84 in the present study.

### 2.3. Intervention

The PMR intervention group had 12 weeks of PMR practice, which followed previously standardized and validated procedures of Bernstein and Borkovec [24] based on a classic muscle relaxation program by Jacobson [15]. This technique involved systematically relaxing the major muscle groups of the body with the goal of physical and mental relaxation. The PMR intervention included twelve 40-minute hospital-based group PMR practice sessions over 12 weeks, once per week. The first session included an introductory group discussion of anxiety and depression in patients with PAH at first. Then the subjects were taught how to relax and contract 16 muscle groups (including muscles of the right hand and forearm, right biceps, left hand and forearm, left biceps, forearm, upper section of cheeks and nose, lower section of cheeks and nose, neck and throat, chest, shoulders and upper part of back, abdominal region and stomach, right thigh, right calf, right foot, left thigh, left calf, and left foot) as previously described [25]. From the second to the twelfth group sessions, only group PMR practice was performed. The average attendance rate for the group PMR sessions was 11.2 ± 3.7 times per patient (range 6–12). Those who participated in ≤8 times of group PMR sessions were excluded from analysis (total *n* = 7, among which *n* = 3 for feeling unnecessary to continue PMR practice because of lack of improvement and *n* = 4 for loss of contact during the intervention). A brochure describing the mechanisms and benefits of relaxation and a relaxation audio CD providing a helpful guide for in-home PMR practice were given to the patients, who were requested to practice PMR at home once per day and record relaxation experiences in a specific form. They were also requested to bring their forms in the group sessions. The in-home PMR practice was reviewed at the start of each weekly group session, permitting discussion of problems and encouragement to practice. According to analysis results of the PMR exercise recording forms, all subjects practiced PMR at home 0.8 times per day on average. The control group received twelve 40-minute sessions of hospital-based mild group stretching and balance exercises over a 12-week duration, one session per week. The first session included an introductory group discussion of anxiety and depression in patients with PAH in addition to group exercise. The average attendance rate for the group exercise sessions was 10.7 ± 3.9 times per patient (range 5–12). Those who participated in ≤8 times of group exercise sessions were excluded from analysis (total *n* = 5, among which *n* = 1 for moving out of down and *n* = 4 for loss of contact during the intervention). The interventions were delivered by the same set of intervention nurses who had received standardized training on PMR. To avoid data bias, care was taken to ensure that PMR and mild group stretching and balance exercises were the only content of the group sessions for the PMR group and the control group, respectively. All intervention nurses were instructed not to personally influence (e.g., encourage or discourage) the patients beyond intended interventions.

### 2.4. Data Collection

All patients were evaluated with HADS and SF-36 within 72 hours before and after the intervention. All patients also underwent a clinical workup including 6-minute walking distance (6MWD) under standardized conditions [8], lung function tests, and cardiopulmonary exercise testing within five days before and after the intervention.

### 2.5. Statistical Analysis

Statistical analyses were performed with SPSS 13.0 for Windows by statisticians who were blinded to the intervention group assignment. For continuous variables, all values were expressed as mean ± SD. Categorical variables were compared with Chi-square tests. Within-group differences in overall/domain QOL, anxiety, and depression scores before and after intervention were tested using paired *t*-tests. Between-group comparisons of the changes in scores after intervention were done with independent *t*-tests. The HADS and QOL scores were also analyzed with repeated measures ANOVA. *P* < 0.05 was considered statistically significant in this study.

## 3. Results

Out of 65 patients with PAH in each group, 55 in the PMR group and 59 in the control group completed the study. There was no significant difference in sociodemographic (Table 1) and clinical (Table 2) characteristics between the two groups at baseline. In addition, no significant differences were found in anxiety, depression, and QOL scores between the two groups at baseline (Table 3). The results indicate that the two groups were comparable at baseline.

Repeated measures ANOVA analyses were performed to evaluate the main effect of time and the PMR intervention in anxiety, depression, and QOL in patients with PAH, respectively. The effect of interaction between time and the PMR intervention was also assessed. *F* values and corresponding *P* values for each test are listed in Table 3. The partial eta squared values, which indicate the percentage of variance in anxiety, depression, or QOL attributable to the effect of time, the PMR intervention, or interaction between time and the PMR intervention, are also listed. As shown in Table 3, repeated measures ANOVA revealed that time had no significant main effect on anxiety, depression, and QOL, indicating that time itself did not have significant impact on psychological health and QOL of patients with PAH. The PMR intervention showed significant main effect as well as interaction with time on anxiety, depression, overall QOL, and QOL-MCS but not QOL-PCS. This indicates that PMR practice was effective in improving psychological health and the mental health components of QOL in patients with PAH and that the effect would strengthen over time. The partial eta squared showed that PMR had the largest effect on the mental health components of QOL (QOL-MCS), followed by anxiety, depression, and overall QOL. As shown in Table 4, the PMR group showed significant improvement in anxiety, depression, overall QOL, and QOL-MCS but not QOL-PCS or 6MWD (*P* < 0.05); in contrast, the control group showed no significant improvement in any of the variables.

Optimal balance between sensitivity and specificity for HADS as a screening instrument was achieved most frequently at a cut-off score of 8+ for both HADS-Anxiety and HADS-Depression [21]. As shown in Table 5, the control group showed no significant changes in distribution of the HADS-Anxiety and the HADS-Depression scores after intervention compared with that at baseline. In contrast, the number/proportion of patients with an anxiety or depression score less than 8 markedly increased in the PMR group after intervention compared with that at baseline. As shown in Table 6, the PMR group showed significant improvement in all QOL mental health domains but not the physical health domains. In contrast, the control group showed no significant improvement in any QOL domain. Both groups showed no significant improvement in lung function tests and cardiopulmonary exercise testing (data not shown).

## 4. Discussion

Depression has been detected in up to 55% in patients with PAH [7]. A recent study has shown that anxiety and depression are frequently detected and significantly correlated with QOL in patients with PAH [8]. In the present study, we demonstrate the effectiveness of PMR as a psychosomatic intervention in anxiety, depression, and QOL in patients with PAH.

By randomized assignment, sociodemographic factors that may affect the PAH patients' anxiety, depression, and QOL status, including age, gender, BMI, religion, education level, economic status, and living status, were comparable between the control group and the PMR group in this study. As mental health problems in pulmonary hypertension patients reportedly are related to the degree of functional impairment [5], we also compared clinical characteristics and found that clinical parameters including 6MWD, WHO functional class, common comorbidities, and PAH-targeted medication and combination therapy were all comparable between the two groups. The results indicate that most potential confounding factors that may affect the PAH patients' anxiety, depression, and QOL status were well balanced between the study groups at baseline. This, combined with blinded analysis of data, increased the reliability of our results.

PMR is a primary method that can be easily learned to achieve relaxation. It is an effective intervention in reducing emotional distress. For example, practice of PMR has been proven to decrease or delay the onset of conditioned symptoms [26]. Regular practice of PMR can also enhance coping ability in a variety of stressful situations [27]. In addition, many empirical studies have found that PMR can enhance feelings of self-control [26, 28, 29]. When PMR is practiced regularly, it provides patients with an intimate familiarity with tension and produces feelings of relaxation throughout the body, which helps to reduce anxiety over time [30]. As a systematic technique used to achieve a deep state of physical and mental relaxation, PMR has been proven effective in reducing anxiety and depression in a variety of conditions including insomnia, asthma, coronary artery bypass surgery, and chemotherapy-induced nausea [10, 16–18]. Based on literature search on PubMed (http://www.ncbi.nlm.nih.gov/pubmed), our study provides the first evidence suggesting that PMR can also effectively improve anxiety, depression, and QOL in patients with PAH. However, PMR only improved the PAH patients' psychological health but not their physical health, since the 6MWD and the QOL-PCS (including physical function, physical role, bodily pain, and general health) were not significantly changed after 12 weeks of PMR intervention. Thus, the therapeutic effects of PMR on patients with PAH were limited to psychological health, particularly, anxiety and depression. PMR, but not the control intervention, significantly decreased the number/proportion of patients with clinically important anxiety or depression (HADS-A or HADS-D scores >8) [21, 31] compared with that at baseline, suggesting that PMR can lead to clinically significant improvement in anxiety and depression in patients with PAH and therefore could be used as a psychosomatic therapy for this patient population.

There are some limitations to the study. (1) The study population was limited to patients with PAH in WHO functional classes I–III [2–4]. We did not include patients in WHO functional class IV based on the consideration that patients with PAH in this particular functional class would be unlikely to keep attending the weekly group PMR sessions due to extremely poor health conditions. (2) This study excluded patients with current mental illness or a family history of mental illness, which may have excluded many patients with PAH who might benefit from the PMR intervention. However, in this first exploratory study on the effectiveness of PMR intervention in PAH patients, we aimed to recruit patients without a history of mental illness so as to exclude potential confounding factors. Based on the findings of this study, future studies involving patients with a wider range of psychological health status will be feasible. In addition, since this study was based on self-report questionnaires, it would be helpful to verify the findings in future studies by a trained clinician using* Statistical Manual of Mental Disorders* (DSM-V) criteria for anxiety and depression [32]. (3) The final follow-up was at the end of the study. Since this was a short-term study, continued effects of PMR practice after completion of the study are an interesting topic for future studies. (4) The clinical workup before and after the intervention included 6MWD, lung function tests, and cardiopulmonary exercise testing but not right heart catheterization. This was due to the following: (a) our pilot study with 50 patients with PAH had not shown any significant change in cardiac catheterization-related parameters, which was corroborated by the present study in which no significant improvement in 6MWD and QOL-PCS was observed after the intervention; (b) there was a balance between funding and the scale of study. (5) Only Han Chinese patients were enrolled in this study, which minimized background noise for the study. However, although Chinese Han population accounts for 90% of the population in China and 19% of global population [33], enrollment of a single ethnicity in this single-center study may limit the generalizability of our findings. Thus, it will be interesting to test the effectiveness of PMR on patients with PAH in other ethnicities in multicenter studies in the future.

In conclusion, our study suggests that PMR practice is effective in improving anxiety, depression, and the mental health components of QOL in patients with PAH. This study provides evidence supporting psychosomatic intervention in the psychological health and QOL of patients with PAH.

## Figures and Tables

**Table 1:** Baseline sociodemographic characteristics of patients.

		Control (*n* = 59)	PMR (*n* = 55)	*P*
Age (years)		53.6 ± 12.2	54.5 ± 13.0	0.79

Age group (years) *n* (%)	<40	7 (11.9)	5 (9.1)	0.98
	40–49	12 (20.3)	12 (21.8)	
	50–59	15 (25.4)	13 (23.6)	
	60–69	14 (23.7)	15 (27.3)	
	≥70	11 (18.7)	10 (18.2)	

Gender *n* (%)	Female	40 (67.8)	41 (74.5)	0.54

BMI		29.4 ± 5.7	30.1 ± 6.3	0.54

Religious *n* (%)	Yes	17 (28.8)	14 (25.5)	0.83
	No	42 (71.2)	41 (74.5)	

Living alone *n* (%)	Yes	21 (35.6)	22 (40.0)	0.70
	No	38 (64.4)	33 (60.0)	

Education level *n* (%)	≤middle school	12 (20.3)	10 (18.2)	0.94
	High school	27 (45.8)	25 (45.5)	
	≥college	20 (33.9)	20 (36.3)	

Economic status *n* (%)	Poor	9 (15.3)	8 (14.5)	0.95
	Fair	24 (40.7)	24 (43.6)	
	Good	26 (44.0)	23 (41.9)	

Smoking status *n* (%)	Current	3 (5.1)	4 (7.2)	0.79
	Former	50 (84.7)	44 (80.0)	
	Never	6 (10.2)	7 (12.8)	

Note: for continuous variables, all values were expressed as mean ± SD and Student's *t*-tests were performed to compare means between the groups. For categorical variables, all values were expressed as *n* (%) and comparisons were performed with Chi-square tests. BMI: body mass index.

**Table 2:** Baseline clinical characteristics of patients.

		Control (*n* = 59)	PMR (*n* = 55)	*P*
PAH diagnosis *n* (%)	Idiopathic	26 (44.1)	27 (49.1)	0.87
	Congenital heart disease	19 (32.2)	16 (29.1)	
	Connective tissue disease	14 (23.7)	12 (21.8)	

PAH therapy *n* (%)	Monotherapy	34 (57.6)	30 (54.5)	0.89
	Dual therapy	20 (33.9)	21 (38.2)	
	Triple therapy	5 (8.5)	4 (7.3)	
	Oxygen therapy	23 (39.0)	24 (43.6)	

PAH-targeted medication	ET receptor antagonists	36 (61.0)	35 (63.6)	0.98
	Phosphodiesterase-5 inhibitors	37 (62.7)	36 (65.5)	
	Prostanoids	10 (16.9)	8 (14.5)	
	Calcium channel blockers	9 (15.3)	9 (16.4)	

Course of disease (years)		7.9 ± 5.2	7.5 ± 5.4	0.65

6MWD (meters)		374.9 ± 136.2	368.2 ± 143.5	0.87

WHO functional class *n* (%)	I	3 (5.1)	2 (3.6)	0.92
	II	24 (40.7)	22 (40.0)	
	III	32 (54.2)	31 (56.4)	

Hypertension *n* (%)		41 (69.5)	37 (67.3)	0.84

COPD *n* (%)		18 (30.5)	19 (34.5)	0.69

Coronary heart disease *n* (%)		15 (25.4)	12 (21.8)	0.67

Type II diabetes mellitus *n* (%)		11 (18.6)	9 (16.4)	0.81

Sleep apnea *n* (%)		9 (15.3)	10 (18.2)	0.80

Note: for continuous variables, all values were expressed as mean ± SD and Student's *t*-tests were performed to compare means between the groups. For categorical variables, all values were expressed as *n* (%) and comparisons were performed with Chi-square tests. PAH: pulmonary arterial hypertension; 6MWD: 6-minute walk distance; WHO: World Health Organization; ET: endothelin; COPD: chronic obstructive pulmonary disease.

**Table 3:** Effects of PMR and time on anxiety, depression, and quality of life (QOL).

Dependent variable	Time			PMR intervention			Time × PMR intervention		
	*F*	*P*	Partial eta squared	*F*	*P*	Partial eta squared	*F*	*P*	Partial eta squared
Anxiety	1.45	.28	.05	13.65	.00	.27	4.17	.04	.18
Depression	1.44	.28	.05	12.84	.00	.25	4.12	.04	.17
QOL-PCS	1.65	.20	.09	1.61	.22	.10	1.56	.23	.08
QOL-MCS	1.52	.25	.06	20.37	.00	.37	7.62	.00	.23
Overall QOL	1.62	.22	.08	9.72	.00	.20	4.05	.04	.15

Note: anxiety, depression, and overall QOL scores were analyzed with repeated measures ANOVA. PMR: progressive muscle relaxation.

**Table 4:** Anxiety, depression, and quality of life (QOL) scores and six-minute walk distance at baseline and after intervention.

	Control (*n* = 59)				PMR (*n* = 55)			
	T1	T2	Δ	*P* ^*^	T1	T2	Δ	*P* ^*^
Anxiety	7.4 ± 3.6	6.9 ± 3.2	−0.5 ± 3.4	.51	7.3 ± 3.5	4.9 ± 2.2	−2.4 ± 2.9^a^	<.01
Depression	7.2 ± 3.8	6.8 ± 3.4	−0.4 ± 3.5	.53	7.1 ± 3.5	4.8 ± 2.4	−2.3 ± 3.1^a^	<.01
QOL-PCS	46.2 ± 20.2	50.2 ± 24.0	4.0 ± 1.9	.38	46.3 ± 21.8	52.5 ± 23.2	6.2 ± 2.0	.17
QOL-MCS	54.8 ± 25.0	59.3 ± 25.2	4.5 ± 2.1	.38	54.9 ± 25.1	69.0 ± 26.3	14.1 ± 4.5^a^	<.01
Overall QOL	50.5 ± 22.62	54.7 ± 24.5	4.2 ± 2.0	.34	50.6 ± 23.6	60.8 ± 24.8	10.2 ± 3.7^a^	.03
6MWD	374.9 ± 136.2	405.6 ± 130.5	30.7 ± 9.5	.40	368.2 ± 143.5	402.5 ± 142.3	34.3 ± 10.6	.21

Note: all values are expressed as mean ± SD. Score improvement within each group is represented by Δ = T2 − T1. T1: baseline; T2: 12 weeks after baseline; PMR: progressive muscle relaxation; QOL-PCS: quality of life-physical component summary score; QOL-MCS: quality of life-mental component summary score. ^*^Paired-sample *t*-test *P* value for T1 versus T2 within each group; ^a^ *P* < 0.05 compared with Δ in the control group.

**Table 5:** Distribution of anxiety and depression scores at baseline and after intervention.

	Control (*n* = 59)				PMR (*n* = 55)		
		T1	T2	*P*	T1	T2	*P*
HADS-anxiety	≤8	17 (29)	21 (36)	0.56	18 (33)	40 (73)	<0.01
	>8	42 (71)	38 (64)		37 (67)	15 (27)	

HADS-depression	≤8	22 (37)	25 (42)	0.71	24 (44)	38 (69)	0.01
	>8	37 (63)	34 (58)		31 (56)	17 (31)	

Note: all values were expressed as *n* (%) and comparisons were performed with Chi-square tests. T1: baseline; T2: 12 weeks after baseline; HADS: Hospital Anxiety and Depression Scale; PMR: progressive muscle relaxation.

**Table 6:** Quality of life (QOL) domain scores before and after intervention.

QOL domains	Control (*n* = 59)				PMR (*n* = 55)			
	T1	T2	Δ	*P* ^∗^	T1	T2	Δ	*P* ^∗^
Physical function	38.3 ± 21.2	41.9 ± 23.2	3.6 ± 1.8	.38	37.9 ± 21.2	42.1 ± 23.0	4.2 ± 1.9	.35
Physical role	37.5 ± 20.7	42.7 ± 24.1	5.2 ± 2.6	.23	37.1 ± 20.5	42.9 ± 22.8	5.8 ± 2.7	.15
Body pain	69.1 ± 26.8	73.3 ± 29.1	4.2 ± 1.9	.42	69.4 ± 27.1	74.1 ± 29.3	4.7 ± 2.4	.63
General health	39.9 ± 18.4	43.0 ± 19.1	3.1 ± 1.5	.37	40.6 ± 19.3	42.9 ± 18.6	2.3 ± 1.1	.51
Vitality	43.3 ± 16.9	47.3 ± 17.3	4.0 ± 1.9	.22	42.8 ± 15.8	54.0 ± 22.5	11.2 ± 3.3^a^	<.01
Social function	59.8 ± 25.3	65.5 ± 25.6	5.7 ± 2.4	.26	60.5 ± 24.9	75.6 ± 27.9	15.1 ± 5.5^a^	<.01
Emotional role	60.3 ± 33.5	66.1 ± 35.2	5.8 ± 2.9	.38	59.1 ± 34.2	74.9 ± 28.0	15.8 ± 6.1^a^	<.01
Mental health	55.7 ± 24.3	58.2 ± 24.0	2.5 ± 1.1	.59	57.3 ± 25.3	71.7 ± 28.3	14.4 ± 4.6^a^	<.01

Note: all values are expressed as mean ± SD. Score improvement within each group is represented by Δ = T2 − T1. T1: baseline; T2: 12 weeks after baseline; PMR: progressive muscle relaxation; QOL-PCS: quality of life-physical component summary score; QOL-MCS: quality of life-mental component summary score. ^*^Paired-sample *t*-test *P* value for T1 versus T2 within each group; ^a^ *P* < 0.05 compared with Δ in the control group.
